# Tracking and characterization of a novel conjugative transposon identified by shotgun transposon mutagenesis

**DOI:** 10.3389/fmicb.2024.1241582

**Published:** 2024-03-26

**Authors:** Jericho Ortañez, Patrick H. Degnan

**Affiliations:** Department of Microbiology and Plant Pathology, University of California, Riverside, Riverside, CA, United States

**Keywords:** *Phocaeicola vulgatus*, Bacteroidota, conjugative transposon, horizontal gene transfer, helix-turn-helix motif, ADP-ribosylglycohydrolase, Tn mutagenesis mobilization method

## Abstract

The horizontal transfer of mobile genetic elements (MGEs) is an essential process determining the functional and genomic diversity of bacterial populations. MGEs facilitate the exchange of fitness determinant genes like antibiotic resistance and virulence factors. Various computational methods exist to identify potential MGEs, but confirming their ability to transfer requires additional experimental approaches. Here, we apply a transposon (Tn) mutagenesis technique for confirming mobilization without the need for targeted mutations. Using this method, we identified two MGEs, including a previously known conjugative transposon (CTn) called *Bo*CTn found in *Bacteroides ovatus* and a novel CTn, *Pv*CTn, identified in *Phocaeicola vulgatus*. In addition, Tn mutagenesis and subsequent genetic deletion enabled our characterization of a helix-turn-helix motif gene, BVU3433 which negatively regulates the conjugation efficiency of *Pv*CTn *in vitro*. Furthermore, our transcriptomics data revealed that BVU3433 plays a crucial role in the repression of *Pv*CTn genes, including genes involved in forming complete conjugation machinery [Type IV Secretion System (T4SS)]. Finally, analysis of individual strain genomes and community metagenomes identified the widespread prevalence of *Pv*CTn-like elements with putative BVU3433 homologs among human gut-associated bacteria. In summary, this Tn mutagenesis mobilization method (TMMM) enables observation of transfer events *in vitro* and can ultimately be applied *in vivo* to identify a broader diversity of functional MGEs that may underly the transfer of important fitness determinants.

## 1 Introduction

Mobile genetic elements (MGEs) are important drivers of bacterial evolution by promoting gene acquisitions that can profoundly affect a bacterial host’s fitness. Known fitness determinants that can be mobilized include those that enable evasion of host immune responses (Rendueles et al., 2018) and antibiotics (Partridge et al., 2018), as well as the ability to intoxicate hosts (Schmidt et al., 1999) and acquire scarce resources in competitive environments (Frye et al., 2021). Furthermore, sequence evidence indicates that extensive interspecies transfer of MGEs among the Bacteroidota, a Gram-negative phylum of bacteria (formerly Bacteroidetes) that is common in the human gut and can represent as much as 80% of the microbiome of some individuals (Wexler, 2007; Smillie et al., 2010; Human Microbiome Project Consortium (HMP), 2012; Coyne et al., 2014; Nayfach et al., 2019). As a result, computational methods to identify, classify, and determine the prevalence of potential MGE activity from DNA sequences have become increasingly sophisticated (Akhter et al., 2012; Ozer et al., 2014; Roux et al., 2015; Johansson et al., 2020). However, given the structural and sequence diversity of MGEs (Osborn and Böltner, 2002), the possibility of inactivating mutations and the presence of cooperative mobilizing elements (Duerkop et al., 2012), confirming the activity of a computationally predicted MGEs generally requires experimental investigation.

Many confirmed MGEs in Bacteroidota have obvious phenotypes that readily enabled the characterization of their functions (e.g., antibiotic resistance) (Schlesinger et al., 2007; Waters and Salyers, 2013; Wood and Gardner, 2015). In contrast, many predicted MGEs lack evidence for obvious functions that would be amenable to a genetic screen (Durrant et al., 2020). Further, the number of predicted MGEs per genome can be quite high, where among *Bacteroides* spp. 20–50% have at least one plasmid (Wexler, 2007) and 80% encode at least one conjugative transposon (CTn; Shoemaker et al., 2001). As such, strategies that avoid 10s or 100 s of targeted mutations to enable screening for mobilization have the potential to accelerate experimental validation of MGE activity and functions.

One such attempt to implement an untargeted approach to identify functional MGEs in *Enterobacteriaceae* was developed by Tansirichaiya et al. (2022). The authors constructed a type of entrapment vector (Gay et al., 1985), pBACpAK, that expresses its tetracycline resistance (Tet^R^) allele when a *cI* repressor gene is disrupted by a MGE (Tansirichaiya et al., 2022). This approach enabled the detection of MGEs integrating into a focal species, or smaller elements replicating within the focal species (e.g., IS elements). However, this system is limited to MGEs that can integrate into the ~0.6 kb *cI* repressor gene and to species within the *Enterobacteriaceae*. These limitations suggest the opportunity for additional untargeted approaches to identify and track functional MGEs. Transposon (Tn) mutagenesis is a commonly used method to generate untargeted genomic mutations and characterize gene functions among all domains of life (Lampe et al., 1996; Kiljunen et al., 2017). We propose combining Tn mutagenesis with screens for horizontal gene transfer (HGT) in bacteria to efficiently assess MGE activity.

Here we evaluate the effectiveness of capturing MGEs by combining *mariner* Tn mutagenesis and HGT screens for a group of human gut-associated bacteria. Through this untargeted Tn mutagenesis mobilization method (TMMM), we successfully tracked the mobilization of two conjugative transposons (CTns), including a novel CTn in *P. vulgatus* ATCC 8482 that we have named *Pv*CTn. Further, this work provides insight into the regulatory mechanisms of *Pv*CTn, most notably the helix-turn-helix motif encoding gene *BVU3433*. We also provide computational evidence for the prevalence of *Pv*CTn-like elements among a panel of human gut-associated bacterial genomes and metagenomes from globally distributed patient samples. The observed mobilization of *Pv*CTn and the widespread presence of genetically diverse *Pv*CTn-like elements in Bacteroidota reinforce the importance of MGEs shaping the human microbiome.

## 2 Materials and methods

### 2.1 Strains, plasmids, culture conditions

All Bacteroidota cultures were grown on Brain Heart Infusion (BHI) agar supplemented with 10% defibrinated horse blood (BHI-HB; Quad Five, Ryegate, MT) and tryptone-yeast extract-glucose broth (TYG) medium with and without agar using an anaerobic chamber (Coy Laboratory Products, Grass Lake, MI) filled with 73% N_2_, 20% CO_2_, and 7% H_2_. *Escherichia coli* with the appropriate plasmid(s) (pSAM, pNBU2, pExchange, pLGB13) were grown on LB. All strains and plasmids are listed in Supplementary Table S1. Ampicillin (100 μg/mL), gentamicin (200 μg/mL), erythromycin (25 μg/mL), tetracycline (2 μg/mL), cefoxitin (20 μg/mL), 5′-fluorodeoxyuridine (FUdR; 20 μg/mL), and anhydrotetracycline (aTC; 100 ng/mL) were added to the media when appropriate.

During conjugations, donors with conjugative plasmids (pNBU2, pExchange, pLGB13) or conjugative transposons (*Pv*CTn) and Bacteroidota recipients were grown overnight in 5 mL LB and TYG medium, respectively, with the appropriate antibiotic(s). Overnight stationary phase *E. coli* strains were then used to inoculate 10 mL subcultures of LB at the following dilutions: 1:200, 1:500, and 1:750 dilutions. *Bacteroides thetaiotaomicron*, *B. ovatus*, and *P. vulgatus* strains were similarly inoculated into TYG but at lower dilutions: 1:25, 1:50, and 1:100. Subcultured *E. coli* S-17 were shaken aerobically at 250 rpm at 37°C. Bacteroidota were subcultured in an anaerobic chamber, stoppered, removed from the chamber, and incubated statically at 37°C. Growth was monitored and cells were pelleted (4,000 ×*g* for 5 min) when they reached an OD_600_ of ~0.4. The supernatants were removed, and cells were resuspended with 1 mL TYG medium and pelleted again. After removing the supernatant, 1 mL TYG medium was used to combine donors and recipients. The combined cells were then spread onto BHI-HB agar. Conjugation plates with *E. coli* donors were incubated aerobically, while dual-Bacteroidota conjugations were incubated anaerobically. Both aerobic and anaerobic conjugations were incubated at 37°C for 24 h. Conjugation masses were then scraped and resuspended in 5 mL TYG medium. The resuspended conjugation masses were then plated onto LB or BHI-HB agar with serial 10-fold dilutions and antibiotic supplement(s). Conjugation efficiencies were calculated using the following equation:


$$\frac{\mathrm{Recipient}\;\frac{\mathrm{CFUs}}{\mathrm{mL}}}{\mathrm{Transconjugant}\;\frac{\mathrm{CFUs}}{\mathrm{mL}}}$$

### 2.2 Effects of peroxide stress on conjugation

Conjugation experiments were performed as described above, however, once donor and recipient Bacteroidota cells were mixed and pelleted after reaching an OD_600_ of ~0.4, the cells were plated on TYG agar where 880 μM H_2_O_2_ was surface spread 30 min before plating when appropriate. The resuspended conjugation masses were plated on BHI-HB agar with serial 10-fold dilutions and antibiotic supplement(s). Conjugation efficiencies were calculated as described above.

### 2.3 Transposon mutagenesis based identification of MGEs

#### 2.3.1 Generating transposon (Tn) mutant libraries

*Bacteroides thetaiotaomicron*, *P. vulgatus*, and *B. ovatus* were mutagenized through conjugation with *E. coli* S17-1 containing the sequencing-adapted *mariner* transposon plasmid (pSAM) containing an erythromycin resistance cassette (Goodman et al., 2009). Conjugations were carried out as above, however, 100 μL aliquots of the entire conjugation masses were plated on 50 BHI-HB plates supplemented with erythromycin and incubated anaerobically at 37°C for 48 h. Colonies were collected and pooled in TYG + 20% glycerol and stored at −80°C in 500 μL aliquots.

#### 2.3.2 Screening Tn libraries for MGEs

Aliquots of Tn mutant library donors (Erm^R^) were then conjugated with Tet^R^ recipient strains of *B. thetaiotaomicron* VPI-5482, *B. thetaiotaomicron* 3731, *Parabacteroides merdae* ATCC 43184, *B. thetaiotaomicron* VPI-5482 ∆CPS, and *B. uniformis* ATCC 8492. To screen for MGE transfers, the conjugation masses were plated onto BHI-HB supplemented with tetracycline and erythromycin. After incubation for 48 h at 37°C, colonies were re-struck for colony purification of the putative transductants or transconjugants. Individual colonies were then grown overnight in liquid TYG supplemented with tetracycline and erythromycin at 37°C for 24 h.

#### 2.3.3 Arbitrary PCR

To map pSAM’s integration sites, arbitrary PCR assay was used as described previously (Goodman et al., 2009). Amplicons were cleaned using a QIAquick PCR Purification Kit (Qiagen, Hilden, Germany) and submitted for Sanger sequencing through the University of California, Riverside Institute for Integrative Genomic Biology (UCR IIGB) core. The sequences were then used as BLASTn search queries against the *B. thetaiotaomicron* (Refseq:NC_004663), *B. ovatus* (Refseq:NZ_AAXF00000000), and *P. vulgatus* (Refseq:NC_009614) reference genomes.

### 2.4 Cloning and mutagenesis (pExchange, pLGB13, pNBU2)

Multiple vector systems were employed to generate the required strains. First, deletion of *BVU3433* mutants was achieved using the *P. vulgatus* specific thymidine kinase (*tdk*) allelic exchange system (Campbell et al., 2020). Briefly, the ~1kbp regions flanking *BVU3433* were individually amplified, then combined and amplified in the splicing by overlap extension (SOE) reaction. All products were amplified using KAPA HiFi Taq MasterMix (KAPA BIOsystems, Wilmington, MA) with the primers listed in Supplementary Table S1. The purified SOE product was then subjected to restriction digestion (NEB) and ligated into pExchange-*tdk*BV using T4 DNA Ligase (NEB).

Second, targeted insertional mutations of tetracycline resistance (*tetQ*) was achieved with pLGB13 which uses erythromycin-aTC counter selection (García-Bayona and Comstock, 2019). We generated a ~ 2 kb SOE product encompassing the intergenic spacer between two co-transcribed hypothetical proteins *BVU3417* and *BVU3418*. However, at the SOE junction within this intergenic spacer, we included a SpeI and a XmaI restriction sites (Supplementary Table S1). In a two-step process, the SOE product was then digested, cleaned and ligated into pLGB13 as described above. Then a *tetQ* (Martens et al., 2008) cassette was amplified and ligated into the SpeI/XmaI restriction sites in the intergenic spacer.

Third, the *BVU3433* complementation construct was generated using pNBU2-*bla-CfxA* (Campbell et al., 2020). We amplified the gene and its native promoter (351 bp upstream of the start codon) and cloned it as described above into the multiple cloning site of the integrative plasmid pNBU2-*bla*-*CfxA*.

All ligation products were transformed into *E. coli* S-17 with electroporation. Individual ampicillin resistant colonies were isolated and purified plasmids were confirmed through PCR and Sanger sequencing. The confirmed allelic exchange and complementation vectors were then conjugated into the appropriate parent stains (e.g., *P. vulgatus* ∆*tdk*) and recombinant merodiploids were selected for on erythromycin. For the deletion and insertion mutants, merodiploids were allowed to loop out. Resolved merodiploids resistant to 20 μg/mL FUdR or 100 ng/μL aTC were isolated and screened by PCR desired mutations.

### 2.5 Measuring growth kinetics of *Phocaeicola vulgatus*

Wild-type and mutant *P. vulgatus* were grown and washed in TYG media. The cells were normalized and diluted to OD_600_ of 0.002 in TYG and dispensed in triplicate into a 96-well plate. Cell growth was measured every 30 min, over 36 h using a BMG Labtech CLARIOstar plate reader. Doubling times were calculated using the least-squares method for growth between 0.05 and 0.12 OD_600_ (*n* = 3).

### 2.6 RNA isolation and RNA-Seq

Samples for RNA-Seq of *P. vulgatus*, *P. vulgatus* ∆*BVU3433*, and *B. thetaiotaomicron Pv*CTn:*tetQ*::∆*BVU3433* were grown overnight in 5 mL TYG medium. Overnight cells were used to inoculate cultures at 10 mL TYG at a final dilution of 1:25 in biological triplicate. Cell growth was monitored and harvested at an OD_600_ of ~0.4. Total RNA was extracted using a lysis buffer (Degnan et al., 2014) and prepared with a Qiagen RNeasy kit and treated with DNA-*free*™ DNA Removal Kit (Invitrogen).

Total RNA was also extracted from *B. thetaiotaomicron* and *P. vulgatus* during conjugation. Conjugation of the two strains was carried out as above. However, after 24 h of growth, the conjugation mass was scraped from the plate surface into TYG and the cells were immediately pelleted and total RNA was extracted as described above.

Library preparation of RNA was completed by following the Illumina (San Diego, CA) Stranded Total RNA Prep, Ligation with Ribo-Zero protocol and using a starting material of 500 ng of total RNA. The only variation from the Illumina protocol is that the volume used for each reagent was reduced by half. The library was submitted to the UCR IIGB core for quality analysis of the multiplexed samples and sequencing on an Illumina NextSeq mid output 75 bp paired end platform.

RNA-Seq read quality was determined through FastQC^1^ and trimmed using trimmomatic (SLIDINGWINDOW:4:15 LEADING:2 TRAILING:2 MINLEN:70) (Bolger et al., 2014). Transcript expression was calculated using Rockhopper with default parameters (McClure et al., 2013) and trimmed reads were mapped to the *P. vulgatus* ATCC 8482 reference genome (Refseq: NC_009614.1), *B. thetaiotaomicron* VPI-5482 (Refseq:NC_004663), and/or *Pv*CTn when appropriate.

### 2.7 cDNA preparation, qPCR

*Phocaeicola vulgatus* ∆*tdk* strains were grown overnight in 5 mL TYG medium. Overnight cells were used to inoculate cultures at 10 mL TYG at a final dilution of 1:25 in biological triplicate. Cell growth was monitored and harvested at an OD_600_ of ~0.4. When appropriate, cells were perturbed by 880 μM H_2_O_2_ and immediately harvested after 30 min of incubation at 37°C. Total RNA was extracted using a lysis buffer (Degnan et al., 2014) and prepared with a Qiagen RNeasy kit and treated with DNA-*free*™ DNA Removal Kit (Invitrogen).

Complementary DNA (cDNA) was generated from 500 ng total RNA using SUPERase•In™ RNase Inhibitor (Invitrogen) and Superscript-II RT (Invitrogen), where the RNA template was eventually degraded by inoculating 1 N NaOH for 30 min at 65°C and neutralized with 1 N HCl. DNA was isolated using a QIAquick spin column (Qiagen) and eluted in 10 mM Tris-Cl, pH 8.5.

Quantification of *BVU3433* gene expression was measured through real-time quantitative PCR (qPCR) using Bio-Rad CFX96 Touch Real-Time PCR Detection System and SYBR Green (KAPA Biosystems) fluorescent dye. The CFX Maestro Software and ∆∆C_q_ method (Bookout et al., 2006) were used to process and calculate differences in *BVU3433* and *16 s rRNA* (Supplementary Table S2) expression. *BVU3366* and *BVU3378* were used as candidate genes to confirm RNA-Seq expression profiles.

### 2.8 Identification of *Pv*CTn family among gut Bacteroidota

The boundaries of *Pv*CTn were predicted using functional gene annotations of the regions surrounding *BVU3433* and alignments of the genomic region with a panel of related *P. vulgatus* (*n* = 13) and *P. dorei* (*n* = 10) strains using Mauve (Darling et al., 2004; Supplementary Table S3). This included functionally characterizing the genomic region using HMMER v3^2^ with trusted cutoffs to search the PFAM v35 (El-Gebali et al., 2019) and TIGRFAM v15 (Haft et al., 2003) databases (Frye et al., 2021). The CTn attachment sites (*attL* and *attR*) were determined using a combination of Mauve alignment inspection, BLASTn and manual sequence alignment.

The putative negative regulator BVU3433 was subsequently used to screen a total of 134 gut Bacteroidota genomes by BLASTp to identify homologs (bit score ratio ≥ 0.3; Supplementary Table S3). The process to identify the boundaries of *Pv*CTn was repeated for each homolog detected as described above, determining its genomic context and if the homolog was a part of an MGE. Identified genomic regions corresponding to putative CTns were then compared using pairwise BLASTn (E value ≤0.0001), filtered for ≥20% percent length aligned (PLA)^3^ and then clustered using MCL (Inflation = 20) (Enright, 2002). Clusters were visualized in Cytoscape (Shannon et al., 2003). Alignments of individual genes were performed using Muscle v3.8.1551 (Edgar, 2004) and maximum likelihood phylogenies were reconstructed using FastTree v2.1.11 (Price et al., 2010).

### 2.9 Regulatory protein and promoter conservation analysis

Examination of the CTn clusters identified a conserved three gene regulatory region including *BVU3433* and genes *BVU3432* and *BVU_RS21835*. We extracted the intergenic region between *BVU_RS21835* and *BVU3433* (and homologs) for alignment and assessment for conserved sequence features. In addition, 250 nt upstream regions of differentially regulated operons were retrieved from *Pv*CTn and conserved genes in related CTns. MEME (Bailey et al., 2015) was used to analyze these regions for conserved sequence patterns that may be involved as regulator binding sites. Searches were performed iteratively using different combinations of promoter regions, number of patterns retrieved (*n* = 5–6), maximum motif widths (*n* = 20–30), and motif distributions (‘zero or one occurrence per sequence’ or ‘any number of repetitions’).

### 2.10 Detection of *Pv*CTn in human gut metagenomes

To identify the frequency of *Pv*CTn-like elements in human gut metagenome samples we employed a marker gene approach. First, nucleotide sequences for two sets of species-specific markers were retrieved from 46 representative genomes in our panel (Supplementary Table S3) corresponding to universally conserved 30S ribosomal protein S5 (*rpsE*) and the *attB* site in pyruvate phosphate dikinase (*ppdK*). Second, 500 bp regions centered on the *attL* and *attR* of known *Pv*CTn-like elements were retrieved. Finally, the terminal 500 bp of *Pv*CTn-like integrases and the entire sequences of *BVU3433* homologs were retrieved. In the end, all markers were ~ 500 bp in size. Then short read metagenomic datasets PRJEB7774 (Feng et al., 2015), PRJEB12449 (Vogtmann et al., 2016) and PRJEB10878 (Yu et al., 2017) from healthy patients and cohorts with colorectal cancer were retrieved from the NCBI SRA database (Supplementary Table S6). The data were quality filtered with Trimommatic as described above and mapped to the 6 marker gene regions using Bowtie2 with the default settings (Langmead and Salzberg, 2012). Conservative estimates of read coverage was measured for each sample based solely on the number of reads spanning the central 24 nt of a given marker gene. This ensured reads spanned the *attB*, *attL* and *attR.* Read coverages were first normalized by the number of reads per sample, then to the read coverage of *rpsE*. Paired reads were also taxonomically classified using Kraken2 (Wood et al., 2019) using the default parameters and the pluspf database (downloaded 17 May 2021).

To evaluate the robustness of this analysis, simulated short read data were generated from the genomes of *P. vulgatus* ATCC 8483 and 14 additional strains with wgsim (−1 100 –2 100 –d 300 –s 100 –N 4000000) part of the SamTools v1.16 package (Danecek et al., 2021) Three groups of ten randomized datasets of 30 million pairs of reads were generated with *P. vulgatus* ATCC 8483 comprising 10, 1% or 0.1% of the sample. The remaining reads in each dataset were randomly selected but divided equally among the other 14 strains. The datasets were then mapped with Bowtie2 against the marker genes and observed and expected read coverages were evaluated as described above.

## 3 Results

### 3.1 Tn mutagenesis enabled the identification of functional MGEs

**Figure 1:**
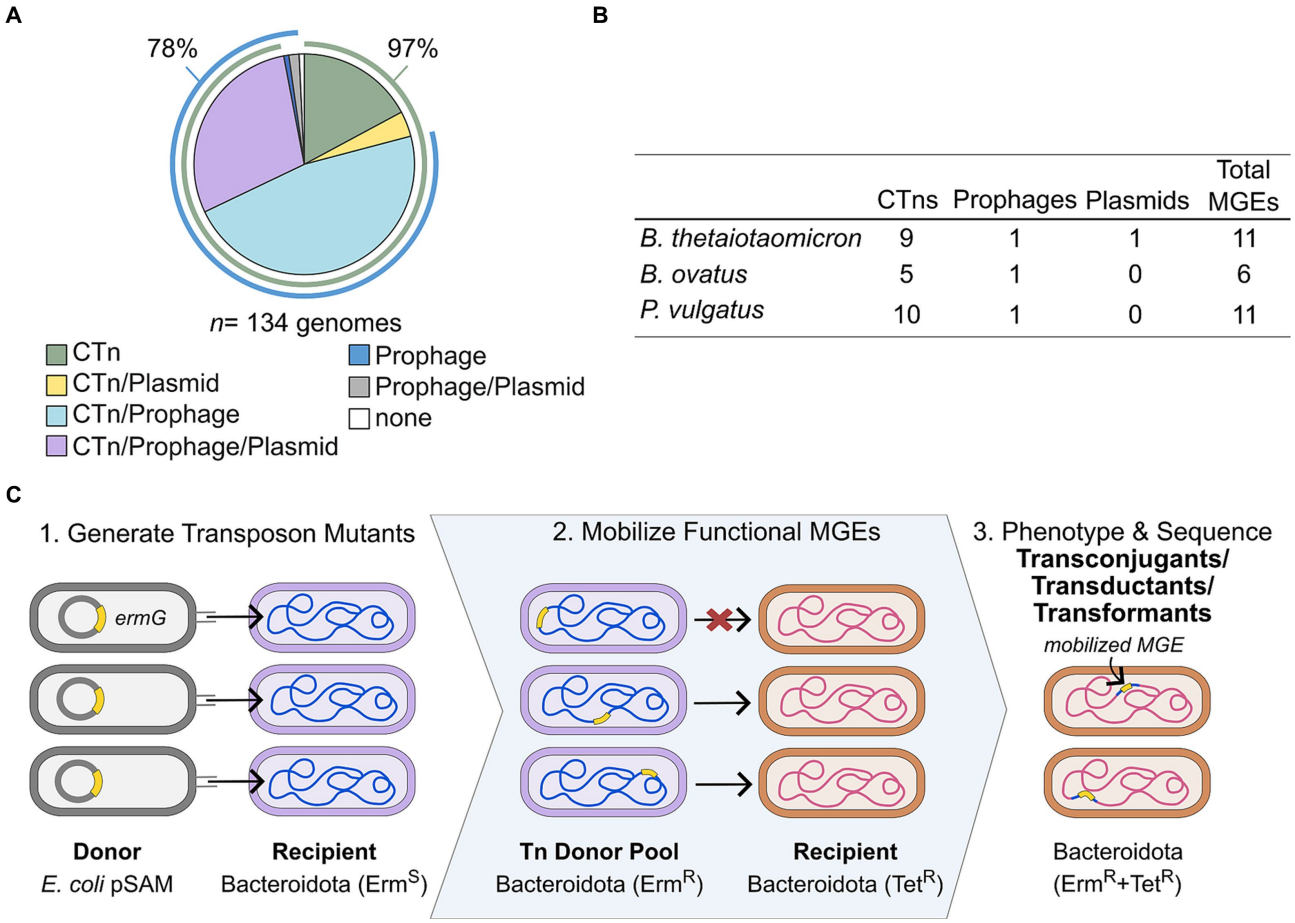
MGEs are common in human gut-associated Bacteroidota. **(A)** The proportions of 134 human gut-associated Bacteroidota with one or more of the computationally predicted MGE element classes (prophage, CTn and plasmid). **(B)** Predicted MGEs counts for the three Bacteroidota species used to generate Tn mutant libraries. **(C)** Schematic of untargeted Tn mutagenesis mobilization method (TMMM) for functional MGE detection.

Computationally predicted mobile genetic elements (MGEs) are common among Bacteroidota genomes, with over 97% of genomes examined having 1 or more predicted type of elements (Figure 1A; Frye et al., 2021). To determine the activity and functionality of these predicted MGEs, we employed an untargeted transposon (Tn) mutagenesis approach. Three prominent gut microbe species with computationally predicted MGEs were selected as donors: *B. thetaiotaomicron* VPI-5482, *P. vulgatus* ATCC 8482, and *B. ovatus* ATCC 8483. Each species has a distinct repertoire of predicted MGEs including CTns and integrated prophages (Figure 1B). Some of these MGEs have been demonstrated to be functional, allowing them to act as positive controls (Reyes et al., 2013; Campbell et al., 2020; Frye et al., 2021).

The first step in the untargeted TMMM (Figure 1C) is to generate Tn libraries using the pSAM INseq vector (Goodman et al., 2009) for the three species. We isolated and pooled ~40,000 independent colonies per Tn library. These Erm^R^ mutant library pools were then used as donors and mated with five Tet^R^ Bacteroidota recipients; *B. thetaiotaomicron* VPI-5482, *B. thetaiotaomicron* 3731, *Parabacteroides merdae* ATCC 43184, *B. thetaiotaomicron* VPI-5482 ∆CPS, and *B. uniformis* ATCC 8492. After mating and selective plating, dually resistant Erm^R^ and Tet^R^ colonies represent putative MGE transfer events (Figure 1C).

The success of the TMMM strategy was mixed. In the mating growth conditions tested here, none of the nine predicted MGEs in *B. thetaiotaomicron* were found to be transferable. However, one *B. ovatus* and one *P. vulgatus* MGE were observed to be mobilizable (Figure 1B). Dually resistant colonies were recovered from matings of *B. ovatus* Tn with *B. thetaiotaomicron* VPI-5482 and *B. thetaiotaomicron* VPI-5482 ∆CPS and colony purified. Using arbitrary PCR (Goodman et al., 2009) we found that all strains screened were transconjugants that acquired *Bo*CTn (16/16), a CTn that encodes a vitamin B_12_ transport locus (Frye et al., 2021). Thus, confirming this strategy for identifying MGE transfer events. For the *P. vulgatus* Tn donor library we initially only isolated dually resistant colonies when *B. thetaiotaomicron* VPI-5482 ∆CPS was used as the recipient. However, all of the isolates (16/16) harbored a single newly acquired and uncharacterized *P. vulgatus* CTn (*Pv*CTn).

### 3.2 Fortuitous detection of a putative repressor of *Pv*CTn activity

**Figure 2:**
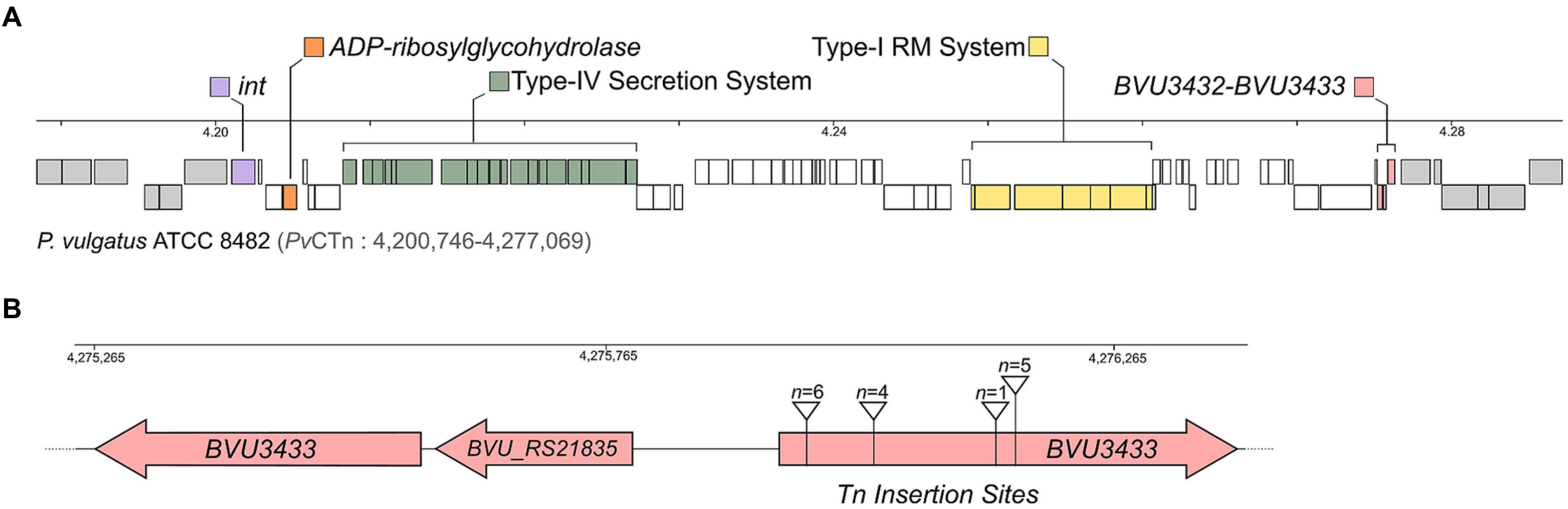
Detection of a mobilizable conjugative transposon *Pv*CTn using Tn mutagenesis. **(A)** Scaled diagram of *Phocaeicola vulgatus* ATCC 8482 genomic region encoding *Pv*CTn and its immediate upstream and downstream regions (genes in grey). Key gene regions described in the text are indicated. Scale bar indicated in megabases (Mb). **(B)** Schematic of the *BVU3432–BVU3433* regulatory region and the four Tn cassette insertion locations identified in *BVU3433* for the 16 screened transconjugants.

*Pv*CTn is ~75 kb with 77 total genes including T4SS machinery, a putative ADP-ribosylglycohydrolase, and a Type I restriction-modification system (Figure 2A; Supplementary Table S2). Examination of the Tn insertion locations for the *B. thetaiotaomicron* VPI-5482 *∆*CPS *Pv*CTn transconjugants revealed that they were all inserted into gene *BVU3433.* This gene encodes a putative 149 amino acid helix-turn-helix (HTH) DNA binding domain protein (PF01381). *BVU3433* has extensive Phyre2 predicted structural homology with diverse transcriptional regulators (e.g., PlcR [2QFC] 99.5% confidence, 91% coverage) (Declerck et al., 2007; Kelley et al., 2015). Further, alignments of the 16 arbitrary PCR products identified a total of 4 independent Tn insertion sites within *BVU3433,* all of which are expected to disrupt its function (Figure 2B). Together, this suggests that the inactivation of *BVU3433* through pSAM mutagenesis de-represses *Pv*CTn conjugation genes, thereby increasing the frequency of *Pv*CTn mobilization.

**Figure 3:**
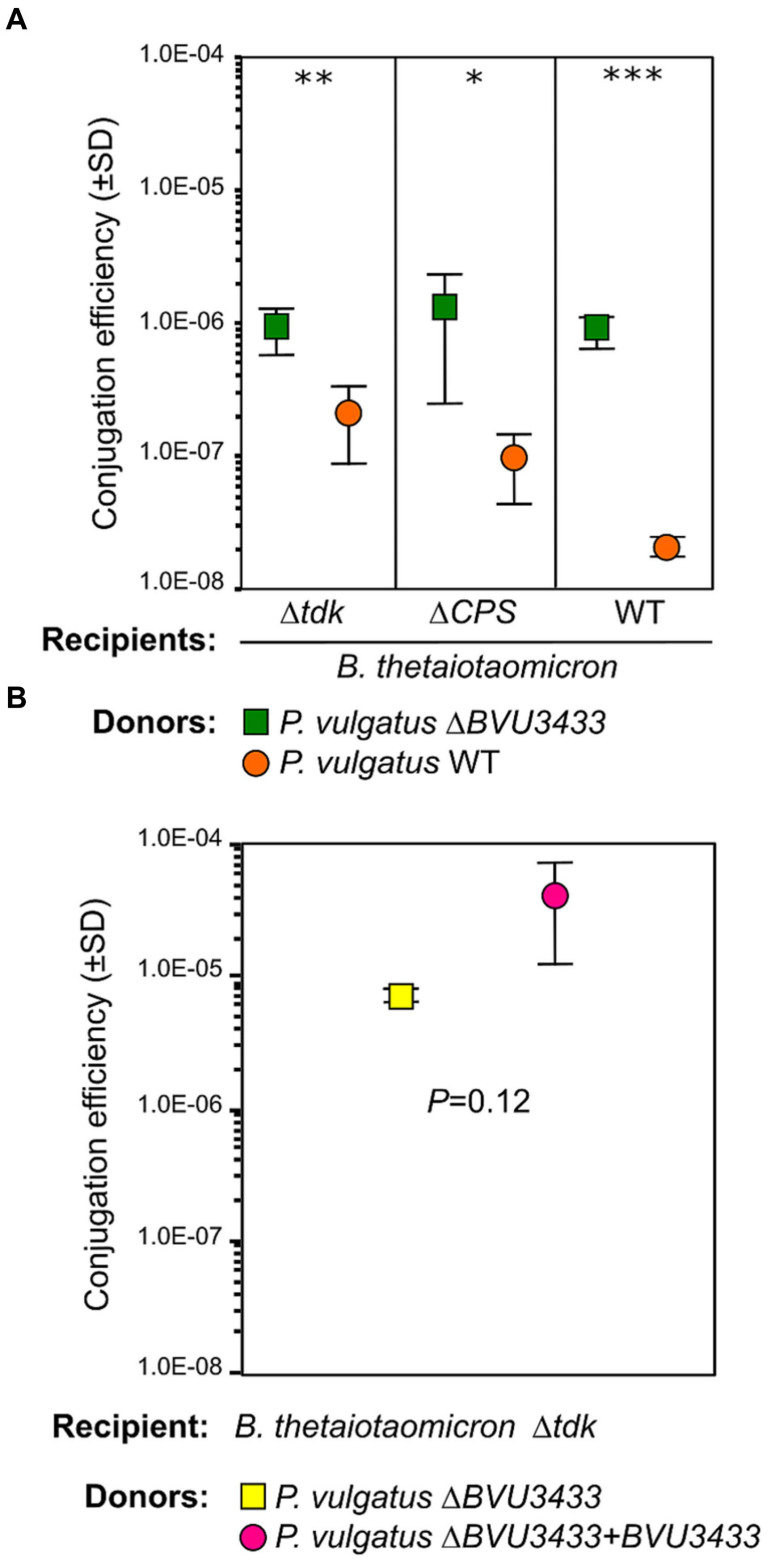
Conjugation efficiency of *Pv*CTn is increased in the *BVU3433* mutant. **(A)** Conjugation efficiencies of *Pv*CTn from WT and ∆*BVU3433 P. vulgatus* donor strains were calculated using three *Bacteroides thetaiotaomicron* recipient strains with two technical replicates for each conjugation combination. This included mutant strains *B. thetaiotaomicron* ∆*tdk* and *B. thetaiotaomicron* ∆*tdk ∆*CPS and their WT parent *B. thetaiotaomicron* VPI-5482. **(B)** Complementation of *BVU3433* in *trans* using pNBU2-*bla*-*CfxA* did not restore lower conjugation efficiencies to *∆BVU3433 + BVU3433* donors. Conjugation efficiencies of donor *P. vulgatus ∆BVU3433* with an empty pNBU2-*bla*-*CfxA* vector or *P. vulgatus ∆BVU3433* pNBU2-*bla*-*CfxA*_*BVU3433* were calculated with *B. thetaiotaomicron* ∆*tdk* recipient cells. Conjugation efficiencies were compared between relevant strains using a one-tailed homoscedastic *t*-test significance (^***^p < 0.001; ^**^p < 0.01; ^*^p < 0.05). All measures represent means ± standard deviation 2–3 technical replicates.

To directly test the function of BVU3433, we hypothesized that deleting *BVU3433* from *P. vulgatus* should increase the conjugation efficiency of *Pv*CTn. We first generated a clean deletion of *BVU3433*, followed by marking *Pv*CTn in the wildtype and deletion background with an antibiotic resistance cassette using allelic exchange resulting in *P. vulgatus* ∆*BVU3433 Pv*CTn:*tetQ* and *P. vulgatus* ∆*tdk Pv*CTn:*tetQ*. We then carried out conjugations with these new donor strains using *B. thetaiotaomicron* VPI-5482 pNBU2_*ermG* and *B. thetaiotaomicron* VPI-5482 *∆*CPS pNBU2_*ermG* as the recipient(s). The conjugation efficiencies of *Pv*CTn ranged from 1.71 × 10^−8^ to 2.11 × 10^−7^ CFUs/mL for *P. vulgatus Pv*CTn:*tetQ* and 8.77 × 10^−7^ – 1.28 × 10^−6^ CFUs/mL for *P. vulgatus Pv*CTn:*tetQ* ∆*BVU3433* depending on the recipient used (Figure 3A). Even though *P. vulgatus ∆BVU3433* has a longer lag phase compared to WT (Supplementary Figure S1A), there were consistently 4.4–51.3-fold higher conjugation efficiencies of the *P. vulgatus* ∆*BVU3433* mutant. We attempted to complement *BVU3433* in *trans* using pNBU2-*bla*-*CfxA* however, no significant difference in conjugation efficiency compared to *P. vulgatus* with only pNBU2-*bla-CfxA* was detected (*p* = 0.12; Figure 3B). Regardless, the *P. vulgatus* ∆*BVU3433* results recapitulate the phenotype detected with the initial Tn mutants.

### 3.3 BVU3433 alters the *Pv*CTn transcriptome

After observing the consistent increase in *Pv*CTn conjugation efficiency of the *BVU3433* mutant we investigated differences in *Pv*CTn WT and *∆BVU3433* transcription through RNA-Seq. We hypothesized that the increase in the mutant *Pv*CTn conjugation efficiency is due to BVU3433 no longer repressing the putative conjugation genes in *Pv*CTn. Therefore, we expected the conjugation genes in *Pv*CTn to be upregulated in the mutant due to the lack of *BVU3433* repression.

**Figure 4:**
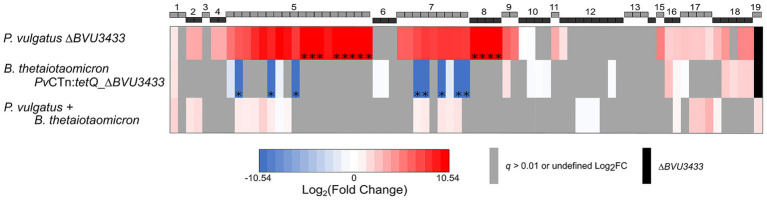
Loss of BVU3433 leads to the upregulation of *Pv*CTn genes. Heatmap represents the log_2_ fold change (log_2_FC) of RNA-Seq gene expression values for *Pv*CTn genes from (i) *P. vulgatus ∆BVU3433,* (ii) *B. thetaiotaomicron Pv*CTn:*tetQ_∆BVU3433,* and (iii) *P. vulgatus* WT x *B. thetaiotaomicron* when compared to *P. vulgatus* WT (only genes with a *q* < 0.01 are shown, genes with *q* > 0.01 and/or undefined log_2_FC values are gray). Black bars represent gene deletions. RNA-Seq for all samples was carried out in biological triplicate. The corresponding gene names for each numbered operon are listed in Supplementary Table S2.

Analysis of the RNA-Seq data revealed that there was indeed an increase in expression throughout *Pv*CTn (Figure 4). In fact, 60% of *Pv*CTn genes (44/73) have a 2-fold or greater upregulation in the mutant. This included operons 4 and 5 that encode the majority of the *Pv*CTn conjugation genes which were expressed an average of 11-fold greater in the *BVU3433* mutant. Using qPCR, we confirmed the upregulation of two candidate operon 5 conjugation genes, one predicted to code for a putative DNA partitioning protein (*BVU3366*) and the other for a conserved protein found in CTns (*BVU3378*). We measured a significant 42 (*p* = 1.58 × 10^−5^) and 23-fold (*p* = 2.28 × 10^−8^) increase in expression in *BVU3366* and *BVU3378*, respectively, in the mutant when compared to WT. This validates our RNA-Seq results (Supplementary Figure S2).

In addition to genes directly linked to the conjugation apparatus, we identified nine other *Pv*CTn-encoded, putative regulatory genes that were differentially regulated by the absence of *BVU3433* (Figure 4; Supplementary Table S4). This includes six putative HTH domain genes: *BVU3415, BVU3420, BVU3426, BVU3429, BVU_RS21735, and BVU3432*. These genes were expressed ~3.5–31.6 fold greater in the *BVU3433* mutant when compared to WT. It is likely that one or more of these HTH genes are involved in the regulation of *Pv*CTn however, the increased expression in the mutant suggests that BVU3433 is the primary regulator repressing these genes. The other three non-HTH regulators include an SOS-response transcriptional repressor (*BVU3423*), a HNH endonuclease (*BVU3427*), and a putative bacterial DNA-binding protein (*BVU3428*). Like the HTH genes, the *BVU3433* mutant has increased gene expression for all three, with a fold change increase ranging from 2.6 to 51.3-fold more compared to WT, further indicating the key role of BVU3433 in *Pv*CTn regulation.

To better understand the effect of BVU3433 on conjugation, RNA from two additional samples were sequenced. The first was from *B. thetaiotaomicron* with a mobilized *Pv*CTn:*tetQ*::∆*BVU3433* from *P. vulgatus* integrated into its genome. Remarkably, we determined that the expression profile of *Pv*CTn:*tetQ*::∆*BVU3433* in *B. thetaiotaomicron* was more like that of the WT *P. vulgatus* than the *BVU3433* mutant. If anything, the expression is lower than WT, with 19% of *Pv*CTn genes (14/73) are at least 2-fold more upregulated in than WT. Expression of operons 4, 5, 7, and 8 are not detected in *B. thetaiotaomicron Pv*CTn:*tetQ*::∆*BVU3433* (Figure 4). The lack of conjugation gene expression needed to produce the T4SS structures encoded in operons 4 and 5 suggests that *Pv*CTn:*tetQ*::∆*BVU3433* in a *B. thetaiotaomicron* recipient likely has reduced conjugation efficiency. Therefore, despite lacking BVU3433, the significant de-repression of genes observed in the mutant *Pv*CTn in one host background (*P. vulgatus*) does not translate to a different host background (*B. thetaiotaomicron*). One possible explanation for this change in expression profile based on the host background may be due to other existing chromosomal or MGE-encoded regulators in *B. thetaiotaomicron* (Figure 1B).

The final sample we sequenced for RNA-Seq, unlike the previous RNA-Seq samples from mid-log phase cells growing in liquid TYG medium, was extracted directly from a mixed lawn of WT *P. vulgatus* and *B. thetaiotaomicron* growing on BHI-HB agar. Since conjugation does not generally occur in liquid cultures, we evaluated the expression of *Pv*CTn during relevant conditions on solid medium. We hypothesized that the conjugation genes on operons 4 and 5 will be upregulated due to the increased likelihood of T4SS structures being formed in these conditions. RNA-Seq data of the *B. thetaiotaomicron* and *P. vulgatus* conjugation mass shows that even when only a small fraction of cells was likely directly conjugating in the mixed population, a slight upregulation of some operon 4 genes and DNA replication genes in operon 8 is observed. However, unlike the WT and mutant *P. vulgatus*, there is a slight downregulation of a cluster of restriction-modification genes in operon 17. Aside from the slight differences in the three operons mentioned, the expression values are like WT *Pv*CTn in *P. vulgatus* (Figure 4A).

### 3.4 H_2_O_2_ decreases BVU3433 expression and increases conjugation efficiency

**Figure 5:**
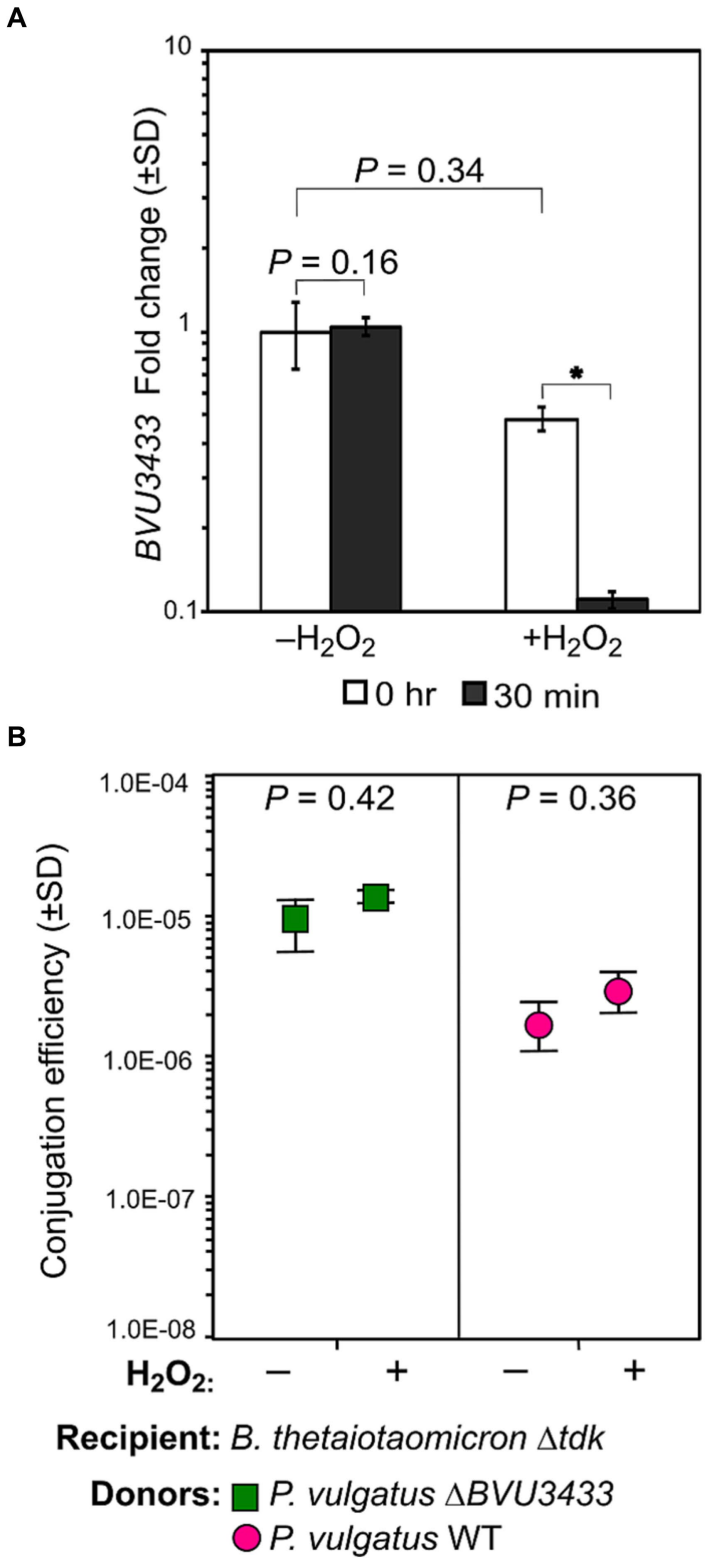
H_2_O_2_ exposure downregulates *BVU3433* expression and increases conjugation efficiency. **(A)** Relative fold change of *BVU3433* was measured in mid-log phase *P. vulgatus* WT treated with H_2_O_2_ or not immediately prior to exposure and after 30 min of incubation at 37°C. RT-qPCR was performed on RNA extracted from biological triplicate cultures for the two treatment groups. **(B)** Conjugation efficiencies of *Pv*CTn from WT and ∆*BVU3433 P. vulgatus* donor strains were calculated using *B. thetaiotaomicron* ∆*tdk* recipients while exposed to H_2_O_2_. Conjugation efficiencies were compared between relevant strains using a one-tailed homoscedastic *t*-test significance from 2–3 technical replicates.

Due to the observed increase in conjugation and de-repression of *Pv*CTn genes in *P. vulgatus* ∆*BVU3433*, we attempted to identify conditions that might influence the expression of *BVU3433*. We hypothesized that stress may be an important driver for the repression of *BVU3433*, therefore, we tested *BVU3433* expression via qPCR of *P. vulgatus* exposed to various sub-inhibitory stress conditions including H_2_O_2_, salinity, antibiotics, taurocholic acid, heat, and UV. We discovered a significant (*p =* 0.015) ~4.4-fold decrease in *BVU3433* expression of H_2_O_2_-exposed *P. vulgatus* when compared to the control group (Figure 5A). Considering this decrease in *BVU3433* expression, we tested how H_2_O_2_-exposure affects conjugation efficiency. We observed an insignificant (*p =* 0.42) 1.73-fold increase in the conjugation efficiency of the H_2_O_2_-exposed WT *P. vulgatus* (Figure 5B) and the *P. vulgatus* ∆*BVU3433* strain followed the same trend with an insignificant (*p* = 0.36) 1.45-fold increase in conjugation efficiency for the H_2_O_2_-exposed mutant (Figure 5B). Together, the qPCR and conjugation efficiency data suggest that subinhibitory H_2_O_2_ stress can increase conjugation efficiency. However, since the conjugation efficiency of both WT and ∆*BVU3433 P. vulgatus* increased with H_2_O_2_ stress, it is possible that H_2_O_2_ not only influences the expression of BVU3433 but is likely to have additional effects on the cell physiology of the donor and/or recipient cells.

### 3.5 *Pv*CTn-like MGEs are common among other Bacteroidota

**Figure 6:**
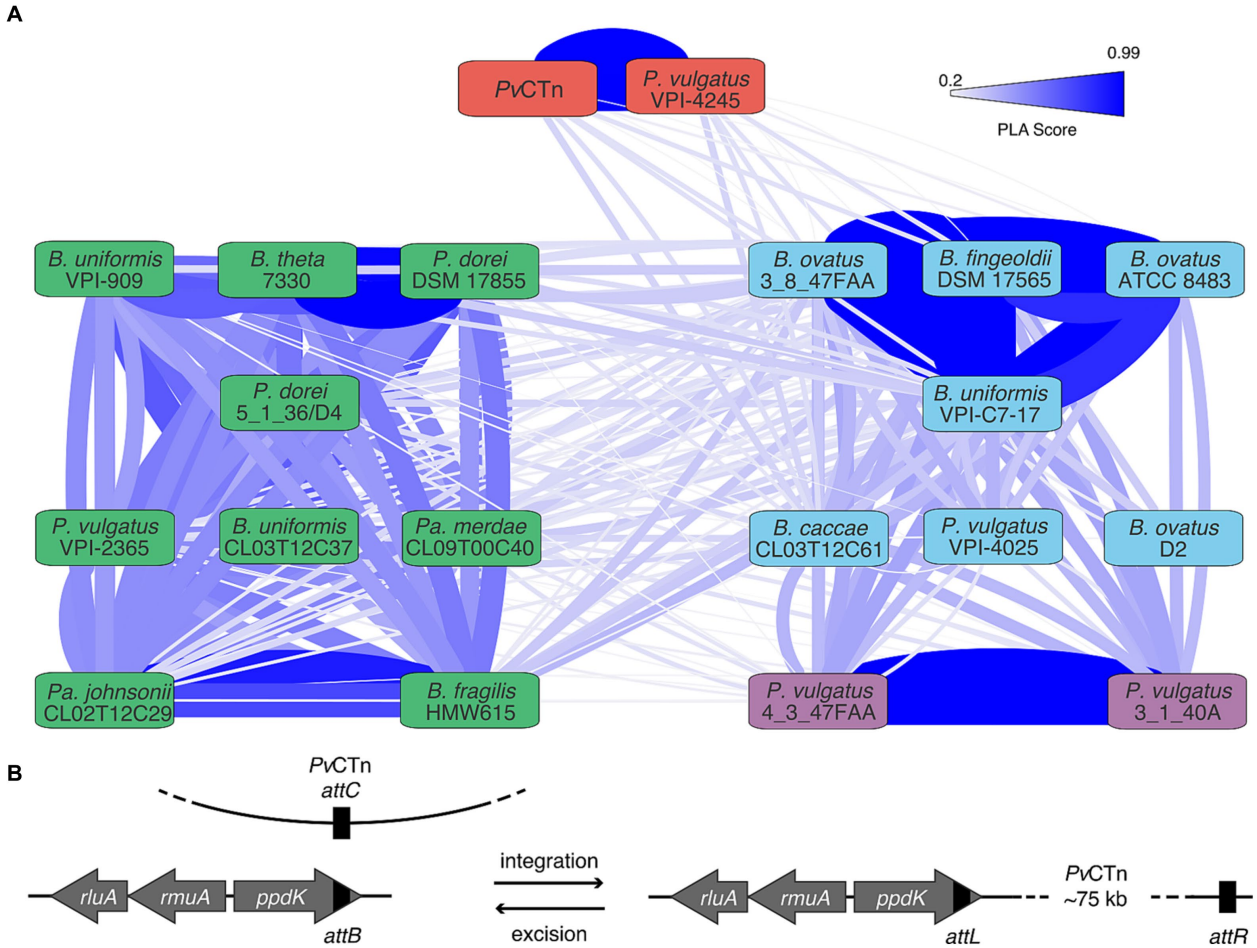
Genetic similarities among *Pv*CTn-like MGEs from human gut microbes. **(A)** Network analysis of DNA sequence similarity among BVU3433-homolog encoding CTns from the human gut microbiome. Four clusters of genome encoded CTn-like elements were identified using MCL based on the percentage of length aligned (PLA) scores. Clusters are indicated by the colored nodes labeled with the strain of origin and edge thickness and color correspond to PLA scores shown in the key (20–99%). **(B)** Schematic of site-specific *Pv*CTn-like MGE insertions in the 3′ end of the strain’s *ppdK* gene. In each case, a stop codon of *ppdK* is regenerated within 2–3 amino acids of expected location after MGE integration.

To determine the prevalence of BVU3433-mediated regulation among gut microbes, we screened 133 other gut microbial genomes for BVU3433 homologs. This search identified 22 putative BVU3433 homologs, 19 of which were encoded by intact CTns found in eight additional species (Supplementary Table S5). The three remaining homologs reside in genomic loci that appear to have experienced deletions or rearrangements leading to the loss of nearly all genes essential for conjugation. Examination of the shared DNA among the 19 CTns content generated four CTn clusters (Figure 6A; Supplementary Figure S3A). Like *Pv*CTn, most encode restriction-modification systems, while others encode putative metal (tellurite) resistance genes (PF02342, PF05099, PF15616), UV protection genes (PF00817, PF11700) and/or protein phosphatases (PF13672).

Despite the divergence in their overall gene content, all 19 CTns share a common integration site at the 3′ end of a conserved three gene cluster *rluA-rmuA-ppdK.* Specifically, we identified the *attB* as the 20 bp motif 5’-GYS GCN CAR GCK GCH RTH GA-3′ within the 3′ end of *ppdK* itself (Figure 6B). Integration of the CTn produces two imperfect direct repeats (*attL* and *attR*) and in each instance regenerates a stop codon within three amino acids of the typical *ppdK* stop codon. Our re-examination of the RNA-Seq read data from our novel *B. thetaiotaomicron Pv*CTn:*tetQ*::∆*BVU3433* transconjugant detected transcriptional readthrough across both the novel *attL* and *attR* sites (Supplementary Figure S3B). These data confirm the preference for this integration location by *Pv*CTn. The conservation of the *attB* is somewhat surprising due to the sequence diversity observed among the predicted integrases (mean = 78.3% ± 9.9% amino acid identity; Supplementary Figure S3C). On further inspection of the *attB* site in the panel of gut microbial genomes we identified another five distinct MGEs of various sizes in 10 genomes that lack *BVU3433* but do encode related integrases (Supplementary Figure S3C).

**Figure 7:**
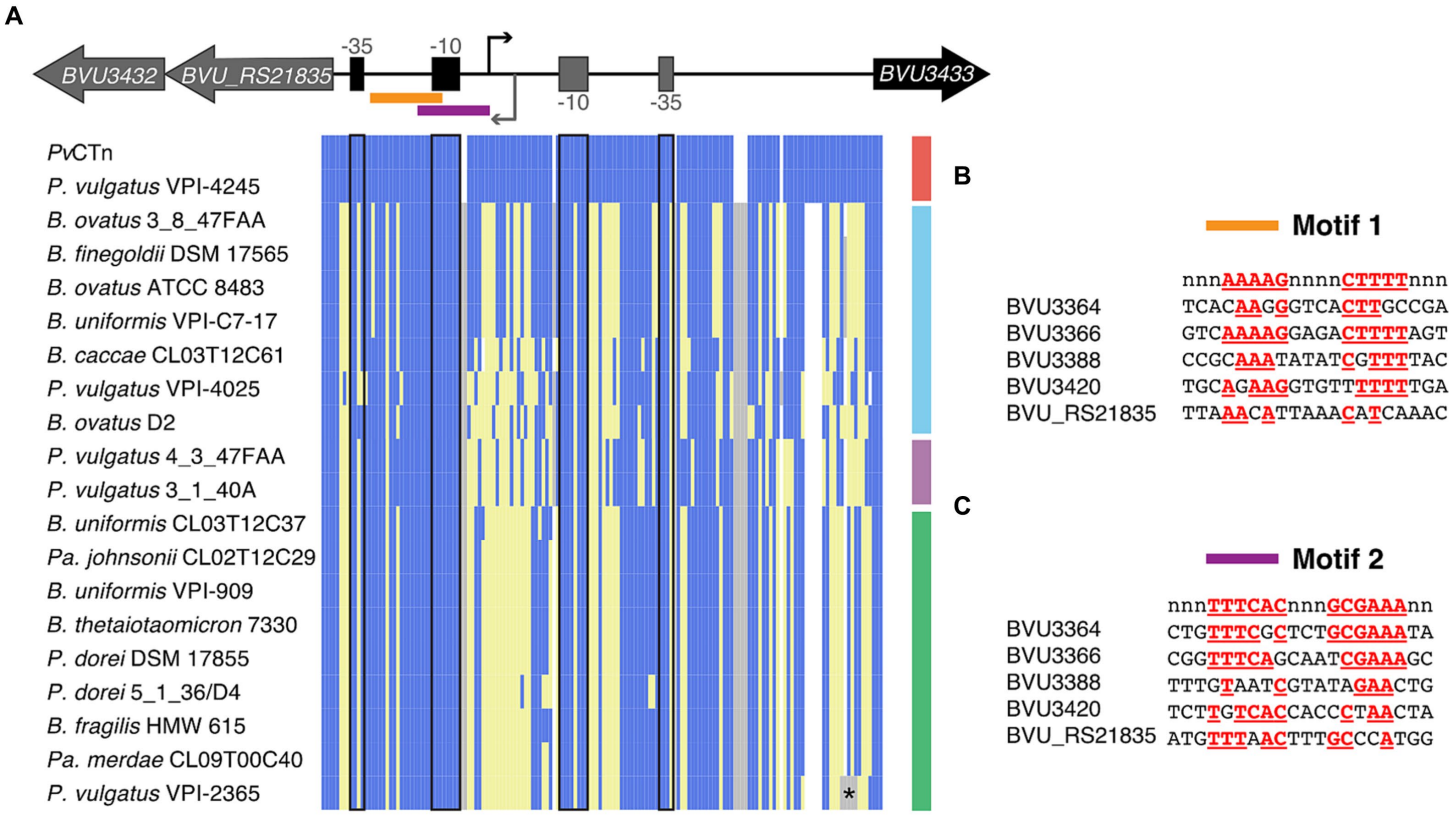
*Pv*CTn-like MGEs encode conserved regulatory regions. **(A)** Sequence alignment of *BVU_RS21835–BVU3433* intergenic region from all 20 *Pv*CTn-like MGEs starting and stopping with the first codon of the indicated gene. The overlapping and conserved RNA polymerase binding sites and transcription start sites are indicated for each divergently transcribed gene. Sites in blue correspond to nucleotides identical to *Pv*CTn, yellow represents alternate nucleotides, white indicates gaps in the alignment, and gray represents inserted nucleotides. The asterisk indicates that *P. vulgatus* VPI-2365 has a hypothetical gene inserted at this location and encoded in the same orientation as *BVU3433*. **(B,C)** Alignments of two conserved motifs with dyad symmetry identified by MEME and found in the upstream regions of five *Pv*CTn operons that are differentially expressed in the ∆*BVU3433* strain and broadly conserved in *Pv*CTn-like MGEs. Majority rule consensus sequence is shown on the top line in red and matching positions are shown for each upstream region.

In addition, we found that all 22 *BVU3433* homologs encode adjacent homologs of *BVU3432* (105AA, *merR*-like HTH domain PF13411) and the small ORF *BVU_RS21835* (63AA, no conserved domains) (Figure 7A). The divergent transcriptional organization of these HTH domain proteins is akin to that of *cI* and *cro* in phage Lambda and similar phages (Degnan et al., 2007). By examining the RNA-Seq transcriptional coverage we were able to identify putative transcriptional start sites and 5′ untranslated regions (5’UTRs) for *BVU3433, BVU_RS21835,* and several differentially regulated operons in *Pv*CTn. We subsequently found that the RNA polymerase binding sites (−10 and −35) of *BVU3433* and *BVU_RS21835* occur within the 5’UTR of the other gene owing to the small size of this intergenic region and are highly conserved among the related CTns (Figure 7A).

Given the broad conservation of the *BVU3432* – *BVU3433* regulatory region among the CTns as well as genes involved in the conjugative apparatus, we computationally searched for conserved motifs that may be involved in regulating conjugation activity. We examined the upstream regions of five genes differentially expressed in ∆*BVU3433* that represent the first gene in the operon and are conserved in ≥16 of the 19 related CTns. This analysis identified conserved features including RNA polymerase binding sites for all genes as well as potential hairpin structures that might act as transcriptional terminators (Figure 7A; Supplementary Figure S3D). The analysis also identified 2 conserved motifs with imperfect dyad symmetry that we observed in all five upstream regions (Figures 7B,C; Supplementary Figure S3D). It is possible that one or both of these motifs act as binding sites for BVU3433 or another *Pv*CTn encoded regulator (e.g., BVU3432) and be responsible for activation or repression of the conjugative apparatus.

### 3.6 Detection of *PvC*Tn in human gut metagenomes

**Figure 8:**
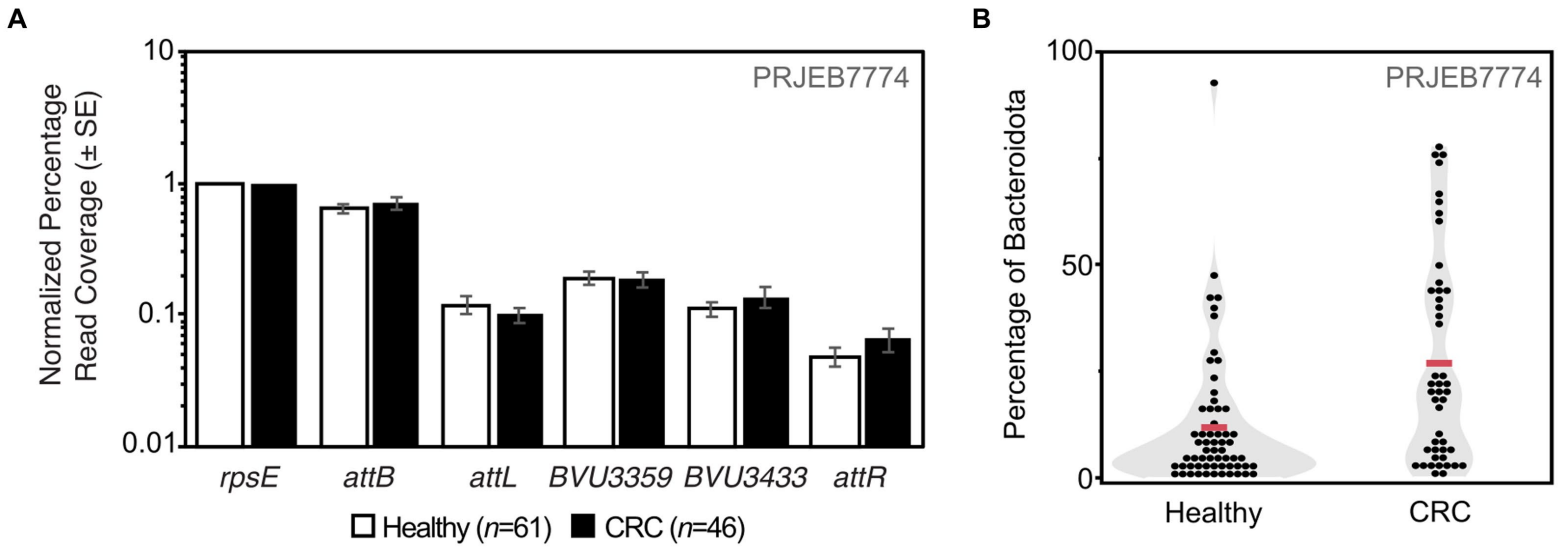
Metagenome detection of *Pv*CTn-like elements. **(A)** Metagenomic short reads from a panel of healthy patients and those with colorectal cancer (CRC) were mapped to the indicated marker genes. Graph shows the average read coverage among samples and error bars represent standard error of the mean (SE). Read coverage was normalized for each sample first by the number of quality filtered short reads and then normalized to gut Bacteroidota *rpsE* gene coverage. **(B)** The same data were also subjected to taxonomic classification using Kraken2 to determine the proportion of Bacteroidota per sample. Individual black dots represent proportion of reads classified as Bacteroidota from the total number of reads that were classified per sample. The average proportion of Bacteroidota among each patient cohort is indicated with the red line (Healthy = 11.7% ± 2.0% SE, CRC = 26.8% ± 3.6% SE).

Using a marker gene approach, evidence for *Pv*CTn-like elements was detected among Bacteroidota species from both healthy patients and those with colorectal cancer (CRC) in 3 geographically distinct studies (Austria, United States, China) (Figure 8A; Supplementary Figures S4A,B). We found sequence evidence for *Pv*CTn-like elements in ~90% of all the patient samples analyzed (300/335). Metagenome sequence reads semi-quantitatively represent both the diversity and abundance of species and strains present in a community sample of isolated cells. As such we can conservatively estimate that ~5–14% of Bacteroidota cells encode a *BVU3433* homolog among the patient sample groups screened. We detected similar coverage ranges indicating the integration of *Pv*CTn-like elements (e.g., ~7–12% *attL* and ~ 2–7% *attR*; Figure 8A; Supplementary Figures S4A,B). The higher prevalence of the integrase (~14–19% *BVU3359*) than *BVU3433* is consistent with our identification of MGEs that share a *BVU3359*-like integrase, but do not encode the *BVU3432–BVU3433* regulatory region (Supplementary Figure S4C). Further, despite the previously described differences in community composition including a greater proportion of Bacteroidota cells (Figure 8B) and immunological status between the healthy patients and those with colorectal cancer (Feng et al., 2015; Vogtmann et al., 2016; Yu et al., 2017) we saw no difference in the frequency of *Pv*CTn-like elements after normalizing for Bacteroidota abundance (Figure 8A).

Analysis of simulated datasets suggests reliable detection of *Pv*CTn when strains with integrated elements account for ≥1% of the population, however, when using the described marker genes and *Pv*CTn encoding strains approach 0.1% detection becomes unpredictable (~50:50; Supplementary Figure S4C). It is likely that such unpredictability could be ameliorated by deeper sequencing coverage. The simulated data, like the actual patient data, also detected a slightly lower than expected coverage of *attL* and *attR* sequences (Supplementary Figure S4C). This is possibly due to a read mapping conflict with the sequence similar *attB* region, but in the patient data it may also be affected by the existence of novel *attL* and *attR* junctions not represented in our marker gene dataset (e.g., novel *Pv*CTn-like MGE integration events).

## 4 Discussion

Bacterial MGEs are known to be significant drivers of bacterial evolution. Therefore, identifying functional MGEs is important for understanding the distribution and exchange of fitness determinants in bacteria. Our study used an untargeted TMMM as a novel approach for tracking functional MGEs in Bacteroidota (Figure 1C). Moreover, this method can be applied to non-Bacteroidota models with established means of Tn mutagenesis including well-known human pathogens (e.g., *Pseudomonas aeruginosa*, *Mycobacterium tuberculosis*, *Vibrio cholerae*) and symbionts (*E. coli*, *Bifidobacterium breve*) (Cain et al., 2020; Dempwolff et al., 2020).

Previous research has established the importance of MGEs in altering bacterial phenotypes, which can have considerable effects on human health (Durrant et al., 2020; Panwar et al., 2023). For instance, the emergence and dissemination of a single carbapenem-hydrolyzing gene (*bla*_NDM_) carried by diverse transposable elements have put the effectiveness of carbapenems, broad-spectrum antibiotics used for treating various bacterial infections, at risk on a global scale (Acman et al., 2022). Moreover, virulence genes have been observed to mobilize from pathogenic to non-pathogenic bacteria (Messerer et al., 2017), underscoring the potential of MGEs to enhance pathogenicity. As the acquisition of MGEs can immediately impact bacterial fitness, discovering novel MGEs could aid the development of techniques to modify microbial communities for improved human health.

### 4.1 Constraints of existing approaches for identifying functional MGEs

Computational methods are effective at identifying potential MGEs in bacterial genomes (Akhter et al., 2012; Ozer et al., 2014; Roux et al., 2015; Johansson et al., 2020). However, predictions alone cannot confirm the ability of an MGE to mobilize, which often requires targeted mutations to confirm or the reliance on capturing chance transfer events in genomic data (Coyne et al., 2014). Our untargeted MGE capture method bypasses the need for directed mutations (Saak et al., 2020). Although a previous study developed transposon-aided capture (TRACA), which allows for the preferential capture of circular extrachromosomal MGEs (i.e., plasmids) (Jones and Marchesi, 2006), it is unlikely to capture elements like *Pv*CTn. On the other hand, entrapment vectors like pBACpAK (Tansirichaiya et al., 2022), if adapted for Bacteroidota hosts, may be able to capture *Pv*CTn. However, this method is reliant upon MGEs inserting into a small region of the recipient pBACpAK vector, which may or may not have sufficient sequence homology for site-specific integrases. In contrast, our method mutagenizes the donor and enables MGEs to integrate at a preferred attachment site within recipient cells.

### 4.2 Tn mutagenesis mobilization method provides insight into CTn regulation

In this study, we successfully identified the mobilization of *Bo*CTn and *Pv*CTn using this Tn mutagenesis method. Curiously, all the *Pv*CTn transconjugants harbored pSAM-mediated disruptions of *BVU3433.* Our subsequent analyses demonstrated a consistent increase in the conjugation efficiency of *P. vulgatus* ∆*BVU3433* mutants compared to WT. While additional Tn insertions likely occurred in T4SS genes or activators of *Pv*CTn mobilization the data suggest that our method may be able to preferentially capture MGEs by disrupting negative regulators of mobilization. However, this is clearly not always the case, as we successfully captured *Bo*CTn which encodes a vitamin B_12_ transporter (Frye et al., 2021) and the Tn cassette integrated into an uncharacterized gene (*BACOVA0479*). Regardless, the range of MGEs captured for each strain will vary based on the functional and regulatory differences in genes essential for mobilization.

Regulation of mobilization of CTns is frequently subject to muti-layer, tight regulation (Salyers et al., 1995; Johnson and Grossman, 2015). CTnDOT, a well-characterized CTn in Bacteroidota, requires tetracycline exposure to trigger a four-step process. This process involves seven regulatory proteins and RNAs in its regulatory cascade for the excision and mobilization of CTnDOT to complete (Waters and Salyers, 2013). Our results identify the critical role of BVU3433 in repressing many *Pv*CTn genes and as a result significantly reducing mobilization. Yet our findings also indicate that *BVU3433* is likely only the first component of the *Pv*CTn regulatory cascade, as nine other *Pv*CTn-encoded regulatory-related genes were upregulated in the absence of *BVU3433*. However, the specific regulatory mechanisms connecting these proteins and the DNA-binding sites of BVU3433 on *Pv*CTn remain to be elucidated. Given the broad upregulation of *Pv*CTn ∆*BVU3433* genes compared to WT *Pv*CTn (Figure 4), we hypothesize that *BVU3433* binds to one or more *Pv*CTn intergenic spacers. Further, we propose that the conserved sequence motifs we identified in the upstream regions of five of the differentially regulated operons represent possible binding sites.

Finally, adding to the complexity of possible regulatory systems within *Pv*CTn, operon 2 of *Pv*CTn contains *BVU3362*, a gene predicted to encode ADP-ribosylglycohydrolase, a class of enzymes commonly involved in post-translational modifications (Mikolčević et al., 2021). In Bacteria, ADP-ribosylation is often linked to the post-translational modification of eukaryotic proteins (Simon et al., 2014). For instance, the Diphtheria toxin produced in *Corynebacterium diphtheriae* inhibits cellular protein synthesis in the host and promotes pathogenicity through the ADP-ribosylation of eukaryotic elongation factor 2 (Bachran et al., 2007). Presently, the regulatory mechanism(s) of BVU3362 within *Pv*CTn is unknown. However, given that ADP-ribosylation has been shown to be involved in *Legionella pneumophila* T4SS effector translocation, it is plausible that BVU3362 may also be involved in the post-translational regulation of the *Pv*CTn T4SS (Amor et al., 2005). This hypothesis will require further investigation to fully understand the role and targets of BVU3362. Overall, this study provides a preliminary characterization of *Pv*CTn’s regulatory mechanism, which is likely to be complex and involve additional layers of transcriptional and translational regulation.

### 4.3 *Pv*CTn-like elements are diverse and globally distributed

Our characterization of *Pv*CTn and *BVU3433* enabled our detection of a diverse group of *Pv*CTn-like elements in human gut-associated bacterial genomes. These conjugative transposons all share a common site-specific integrase and attachment site (*attB*) along with the *BVU3432-BVU3433* regulatory region, and genes involved in the T4SS. While none encode known antibiotic resistance genes, several encode metal and UV resistance genes that may contribute to the fitness of their hosts. Further, virtually all the CTns encode restriction modification systems which may have consequences for further mobile DNA acquisitions by their hosts.Individual bacterial genomes are generally crucial for identifying host-MGE pairs, but they represent only a fraction of species and strain diversity that exists in human guts globally. As such we interrogated the prevalence of *Pv*CTn-like elements in human gut metagenomes (Feng et al., 2015; Vogtmann et al., 2016; Yu et al., 2017), and found they were widespread in ~90% of patient samples. Together these results suggest *Pv*CTn-like elements contribute to genetic and functional diversity of human gut microbes.

## 5 Conclusion

In this study, we have successfully demonstrated a new and effective method for capturing MGEs from gut microbes. Although our approach did not detect mobilization of all the predicted MGEs, it expands the currently available methods for MGE identification and offers a potential strategy for numerous other bacteria. Our method enabled us to confirm *Pv*CTn functionality and identify a conserved conjugation repressor protein. Further, *Pv*CTn represents one member of a diverse family of elements that can be detected in patient samples from around the globe. Further, these *Pv*CTn-like elements may indirectly contribute to human health as studies have highlighted the distribution of *P. vulgatus* and its sister species *P. dorei* in the gut microbiome as a critical determinant in the efficacy of immune checkpoint blockade therapy in advanced melanoma patients (Usyk et al., 2021) and the development of coronary artery disease (Yoshida et al., 2018). And strains of these two species encode 8 of the 20 *Pv*CTn-like elements we identified. Overall, our findings demonstrate the potential of our method for the discovery of novel MGEs and provide insights into the prevalence and distribution of *Pv*CTn-like elements in human gut-associated bacteria.

## Data availability statement

The original contributions presented in the study are publicly available. This data can be found here: NCBI BioProject, accession PRJNA983822.

## Author contributions

JO: conceptualization, methodology, lab work, manuscript writing and reviewing, and data analysis. PD: conceptualization, methodology, developed software, manuscript writing and reviewing, and data analysis. All authors contributed to the article and approved the submitted version.

## Footnotes

^1^ https://www.bioinformatics.babraham.ac.uk/projects/fastqc/

^2^ hmmer.org

^3^ https://doi.org/10.5281/zenodo.5138177
