# Supplementary material for: Tracking and characterization of a novel conjugative transposon identified by shotgun transposon mutagenesis
Source: Front Microbiol. 2024 Mar 26;15:1241582. doi: 10.3389/fmicb.2024.1241582 (PMC11005914; doi:10.3389/fmicb.2024.1241582)
Supplement: Supplementary file 7 [file Data_Sheet_1.PDF]

Supplementary Material

Tracking and Characterization of a Novel Conjugative Transposon Identified by Shotgun Transposon Mutagenesis

Jericho Ortañez, Patrick H. Degnan*

*** Correspondence:**
Patrick Degnan
patrick.degnan@ucr.edu

1 Supplementary Figures

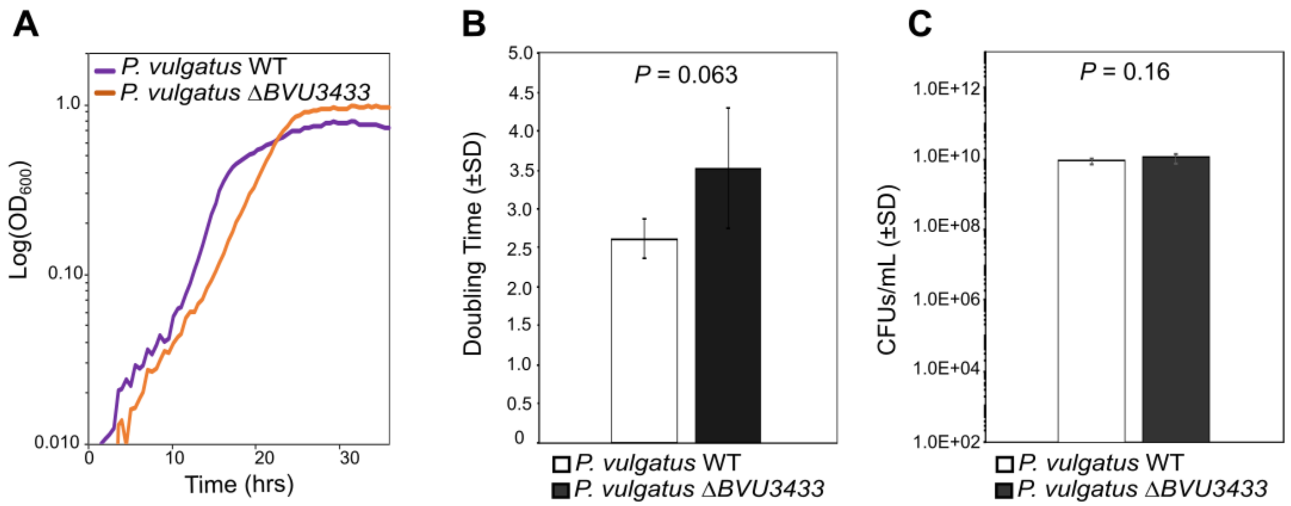

Figure S1. ***P. vulgatus WT*** **and** ***P. vulgatus*** **ΔBVU3433 growth and doubling times. (A)** Representative growth curves in TYG media optical densities (OD_{600}) were recorded every 30 minutes. Curves are averages from technical replicates ($n = 3$). **(B)** Doubling times were calculated using the least-squares method for growth between 0.05 and 0.12 OD_{600} ($n = 3$). **(C)** Colony forming units (CFUs) were calculated for the inocula used in **A**. Both **B** and **C** used a one-tailed homoscedastic *t*-test to test significance.

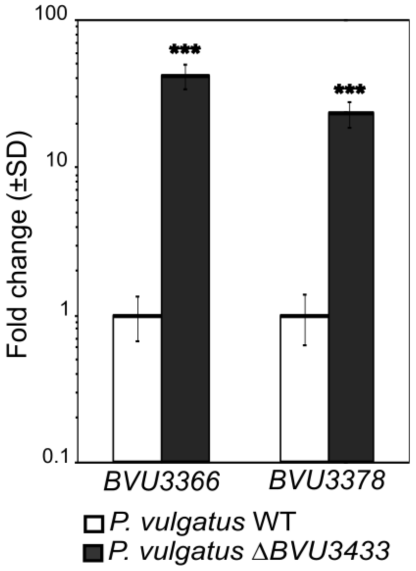

Figure S2. Candidate conjugation genes are upregulated in *P. vulgatus* Δ*BVU3433*. Relative fold change was calculated for representative conjugation genes *BVU3366* and *BVU3378* by RT-qPCR comparing expression in *P. vulgatus* WT versus *P. vulgatus* Δ*BVU3433* during mid-log phase growth in TYG (one-tailed homoscedastic *t*-test to test significance; ***, $P < 0.001$; **, $P < 0.01$; *, $P < .05$).

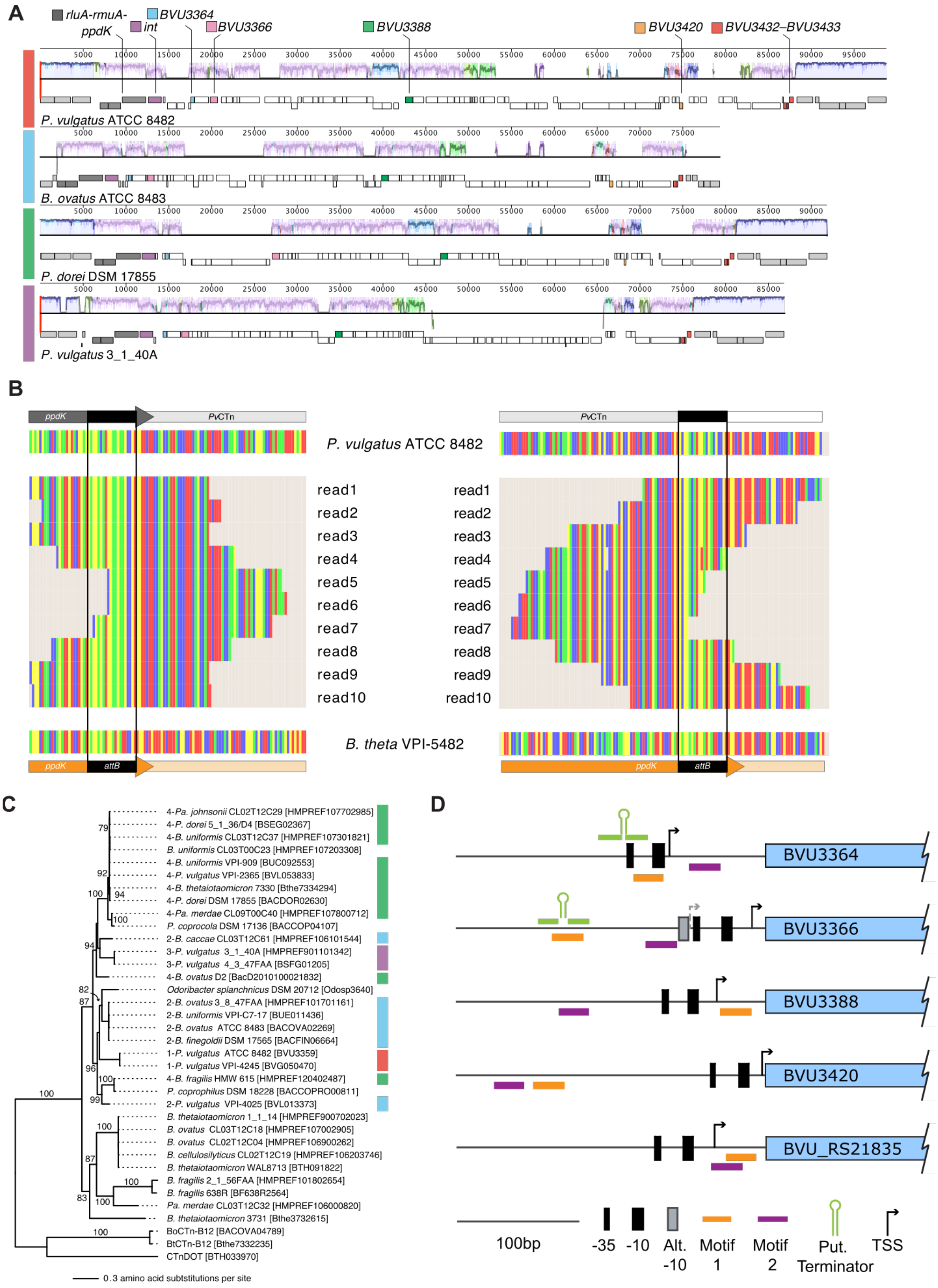
A
rluA-rmuA-ppdK
int
BVU3364
BVU3366
BVU3388
BVU3420
BVU3432–BVU3433
P. vulgatus ATCC 8482
B. ovatus ATCC 8483
P. dorei DSM 17855
P. vulgatus 3_1_40A
B
ppdK
PvCTn
P. vulgatus ATCC 8482
read1
read2
read3
read4
read5
read6
read7
read8
read9
read10
B. theta VPI-5482
attB
C
4-Pa. johnsonii CL02T12C29 [HMPREF107702985]
4-P. dorei 5_1_36/D4 [BSEG02367]
4-B. uniformis CL03T12C37 [HMPREF107301821]
B. uniformis CL03T00C23 [HMPREF107203308]
4-B. uniformis VPI-909 [BUC092553]
4-P. vulgatus VPI-2365 [BVL053833]
4-B. thetaiotaomicron 7330 [Bthe7334294]
4-P. dorei DSM 17855 [BACDOR02630]
4-Pa. merdae CL09T00C40 [HMPREF107800712]
P. coprocola DSM 17136 [BACCOP04107]
2-B. caccae CL03T12C61 [HMPREF106101544]
3-P. vulgatus 3_1_40A [HMPREF901101342]
3-P. vulgatus 4_3_47FAA [BSFG01205]
4-B. ovatus D2 [BacD2010100021832]
Odoribacter splanchnicus DSM 20712 [Odosp3640]
2-B. ovatus 3_8_47FAA [HMPREF101701161]
2-B. uniformis VPI-C7-17 [BUE011436]
2-B. ovatus ATCC 8483 [BACOVA02269]
2-B. finegoldii DSM 17565 [BACFIN06664]
1-P. vulgatus ATCC 8482 [BVU3359]
1-P. vulgatus VPI-4245 [BVG050470]
4-B. fragilis HMW 615 [HMPREF120402487]
P. coprophilus DSM 18228 [BACCOPRO00811]
2-P. vulgatus VPI-4025 [BVL013373]
B. thetaiotaomicron 1_1_14 [HMPREF900702023]
B. ovatus CL03T12C18 [HMPREF107002905]
B. ovatus CL02T12C04 [HMPREF106900262]
B. cellulosilyticus CL02T12C19 [HMPREF106203746]
B. thetaiotaomicron WAL8713 [BTH091822]
B. fragilis 2_1_56FAA [HMPREF101802654]
B. fragilis 638R [BF638R2564]
Pa. merdae CL03T12C32 [HMPREF106000820]
B. thetaiotaomicron 3731 [Bthe3732615]
BoCTn-B12 [BACOVA04789]
BtCTn-B12 [Bthe7332235]
CTnDOT [BTH033970]
0.3 amino acid subtstitutions per site
D
BVU3364
BVU3366
BVU3388
BVU3420
BVU_RS21835
100bp
-35
-10
Alt. -10
Motif 1
Motif 2
Put. Terminator
TSS

Figure S3. Structural and functional conservation of *Pv*CTn-like MGEs from human gut microbes. (**A**) Sequence alignment of representative integrated *Pv*CTn-like MGEs from the four identified clusters. Nucleotide similarity plots are shown above each scaled representation of genes present on the leading and lagging strands. Genes flanking each integrated MGE are shown in gray with the *attB* insertion site in *ppdK* shown in dark gray. The integrase gene and the five differentially expressed and conserved genes are marked with their corresponding colors. (**B**) Alignments of RNAseq short reads confirm integration of *Pv*CTn in the *ppdK* gene of a *B. thetaiotaomicron* VPI-5482 transconjugant. Ten representative reads from the *attL* and *attR* region of *Pv*CTn span the indicated attachment site and continue into the *ppdK* chromosomal region of *B. thetaiotaomicron* and not that of the donor *P. vulgatus*. (**C**) Maximum likelihood phylogenetic reconstruction of *ppdK-attB* inserted MGE integrase proteins. Despite the most divergent ingroup sequences having as little as 42% amino acid identity, all appear to site specifically integrate into *ppdK*. Integrases from a *Pv*CTn-like MGE are marked with a colored bar that corresponds to clusters in Figure 6. FastTree estimated bootstrap values ≥50% are shown adjacent to the appropriate node. Integrases from CTnDOT and vitamin B_{12} transporter encoding CTns were used as outgroups. (**D**) Diagrams of the upstream regions of the five differentially expressed and conserved genes with identified conserved regulatory motifs. Size and spacing of features indicated in the key are to scale.

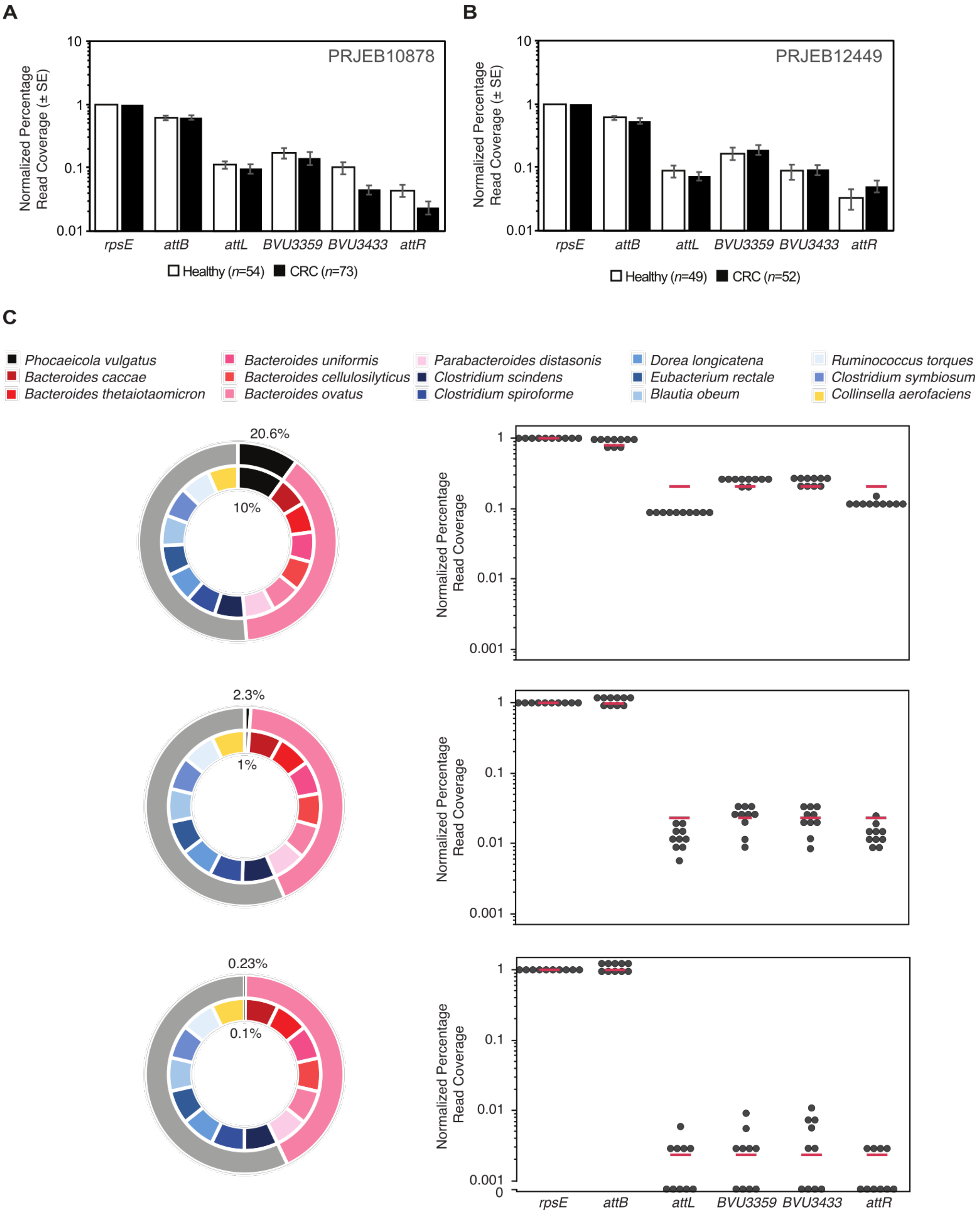

Figure S4. Metagenome detection of *Pv*CTn-like elements in clinical and simulated datasets. (**A, B**) Metagenomic short reads from two additional panels of healthy patients and those with colorectal

cancer (CRC) were mapped to the indicated marker genes. Graphs show read coverage normalized first by number of quality filtered short reads and then normalized to gut Bacteroidota *rpsE* gene coverage. Standard error bars are shown. (**C**) Simulated read data were generated from 15 indicated genomes, then randomly selected for three groups of 10 datasets with 10%, 1% or 0.1% *P. vulglatus* represented. Inner circles of doughnut plots indicate the proportion of each species according to the key and outer circles indicate the proportion of *P. vulglatus* relative to the other Bacteroidota present in the sample (e.g., expected *rpsE* read coverage). Plots at right show normalized read coverage detected for each marker gene from each randomized dataset as a black point. Expected coverage is shown for each marker as a red line. Genomes for the following bacterial strains were used *P. vulgatus* ATCC 8482, *B. caccae* ATCC 43185, *B. thetaiotaomicron* VPI-5482, *B. uniformis* ATCC 8492, *B. cellulosilyticus* WH2, *B. ovatus* SD_CC_2a*, Pa. distasonis* ATCC 8503, *Clostridium scindens* ATCC 35704, *C. spiroforme* DSM 1552, *Dorea longicatena* DSM 1381, *Eubacterium rectale* ATCC 33656, *Blatuia obeum* ATCC 29174, *Ruminococcus torques* ATCC 27756, *C. symbiosum* ATCC 14940, *Collinsella aerofaciens* ATCC 25986.
